# A proportional odds model of risk behaviors associated with recurrent road traffic crashes among young adults in Kuwait

**DOI:** 10.1186/s12874-021-01497-2

**Published:** 2022-01-14

**Authors:** Saeed Akhtar, Eisa Aldhafeeri, Farah Alshammari, Hana Jafar, Haya Malhas, Marina Botras, Noor Alnasrallah

**Affiliations:** Department of Community Medicine and Behavioural Sciences, Faculty of Medicine, Kuwait University, P.O. Box 24923, 13110 Safat, Kuwait

**Keywords:** Recurrent road traffic crash, Driving behaviours, Proportional odds model, Young drivers

## Abstract

**Background:**

The aims of this cross-sectional study were to i) assess one-year period prevalence of one, two, three or more road traffic crashes (RTCs) as an ordinal outcome and ii) identify the drivers’ characteristics associated with this ordinal outcome among young adult drivers with propensity to recurrent RTCs in Kuwait.

**Methods:**

During December 2016, 1465 students, 17 years old or older from 15 colleges of Kuwait University participated in this cross-sectional study. A self-administered questionnaire was used for data collection. One-year period prevalence (95% confidence interval (CI)) of one, two, three or more RTCs was computed. Multivariable proportional odds model was used to identify the drivers’ attributes associated with the ordinal outcome.

**Results:**

One-year period prevalence (%) of one, two and three or more RTCs respectively was 23.1 (95% CI: 21.2, 25.6), 10.9 (95% CI: 9.4, 12.6), and 4.6 (95% CI: 3.6, 5.9). Participants were significantly (*p* < 0.05) more likely to be in higher RTCs count category than their current or lower RCTs count, if they habitually violated speed limit (adjusted proportional odds ratio (pOR_adjusted_) = 1.40; 95% Cl: 1.13, 1.75), ran through red lights (pOR_adjusted_ = 1.64; 95%CI: 1.30, 2.06), frequently (≥ 3) received multiple (> 3) speeding tickets (pOR_adjusted_ = 1.63; 95% CI: 1.12, 2.38), frequently (> 10 times) violated no-parking zone during the past year (pOR_adjusted_ = 1.64; 95% CI: 1.06, 2.54) or being a patient with epilepsy (pOR_adjusted_ = 4.37; 95% CI: 1.63, 11.70).

**Conclusion:**

High one-year period prevalence of one, two and three or more RTCs was recorded. Targeted education based on identified drivers’ attributes and stern enforcement of traffic laws may reduce the recurrent RTCs incidence in this and other similar populations in the region.

## Background

Road traffic crashes (RTCs) claim more than 1.2 million lives each year and as many as 50 million are injured or disabled worldwide [1]. Most affected are young adults – economically productive age-group (15 to 44), which accounts for 59% of global RTCs-related fatalities. RTCs-related public health burden results from high frequency of deaths and disabilities that annually cost governments approximately 3% of gross domestic product (GDP) [2, 3]. Despite these colossal losses in terms of human and economic resources, action to combat this global pandemic of shocking proportion has been insufficient. Road traffic related deaths are likely to increase to an estimated 2.4 million per year by 2030, if the present trends continue unabated [4].

In the World Health Organization (WHO) Eastern Mediterranean and African Region, the road safety statistics are even more alarming with the highest death rate of 32.2 per 100,000 population [5]. Kuwait has one of the highest car ownership rates (411 per 1000 population) in the world [6]. Road traffic injuries (RTIs) are consistently the second most frequent cause of death and the number one cause of death among youth with an average age at death between 21 and 30 years in Kuwait [7, 8]. Furthermore, there is a 13.1% increase in RTIs-related deaths from 2005 to 2006 in Kuwait [8]. Poor driving behavior encompassing nonuse of seats belt, speeding, etc., and the lack of enforcement of traffic regulations are believed to be the main causes of the unsafe driving environment [9, 10]. Furthermore, elsewhere, recurrent of RTCs rate as high as 25% within a year of first RTC among young drivers has been reported [11]. Moreover, this repetitive engagement in RTCs among young drivers has been attributed to human factors including cognitive impairment, sensation taking behaviours including speeding, and specific traffic environment shaped by casual traffic laws enforcement, frequent use of cell phone while driving, eating/ drinking while driving, low seatbelt-use among drivers [11, 12]. However, there is a paucity of the comparable published data on such risk behaviours among young drivers with propensity for involving in recurrent RTC in the Middle Eastern countries specifically in Kuwait.

Investigators in medicine and allied disciplines often seek to the estimate the disease risk measured initially on interval scale (*e.g.,* birth weight, cholesterol level, blood pressure) or originally recorded on ordinal scale (pain or injury as none, mild, moderate or severe) by categorizing it into multiple binary response variables. These binary response variables are used to compute the effect estimates (*i.e.,* risk ratio or odds ratio). This approach of using multiple binary logistic regressions models instead of employing a holistic approach by using an ordinal logistic regression model yields less efficient parameter estimates with an overall more unexplained error [13]. We demonstrated the use a proportional odds model to compute the effect estimate using an all-inclusive approach when the adverse health outcome has multiple ordinal categories, which quite frequently are encountered in epidemiology and other allied fields.

We recently reported one-year period prevalence and factors associated with one or more crashes among young adults in Kuwait [4]. However, binary logistic regression used to identify the factors associated with RTCs status (*i.e.,* ≥ 1 RTCs vs. 0) in that study did not consider the ordinal nature of the RTCs count. As noted above, binary categorization of a natural ordinal variable can result in the loss of information with less efficient effect estimate [13]. Ordered logistic regression takes the natural ordering of RTCs count to evaluate the effect of different driving behaviours on RTCs count. Therefore, the aims of this cross-sectional study were to i) assess one-year period prevalence of one, two, three or more road traffic crashes (RTCs) as an ordinal outcome and ii) identify the drivers’ characteristics associated with this ordinal outcome among young adult drivers with propensity to recurrent RTCs in Kuwait.

## Methods

### Study design, setting and participants

The study procedures, including a description of study design, setting, and study population have been described previously [4], and are briefly outlined here. Kuwait is a small country in the Middle East with a total area about 18,000 km^2^. Kuwait is located at the north-west corner of the Arabian Gulf. Kuwait shares border with Iraq on the north-west and has a common border with Saudi Arabia on the south. Kuwait has petroleum-based economy, oil and gas account for nearly 60% of the GDP and about 95% of export revenues. Total population of Kuwait is 4.3 million, 68% of which is non-Kuwaiti migrant workers and/ or their dependents. Non-Kuwaiti migrants contribute 80% of the labour force in the country [14]. Non-Kuwaiti male among other also take up the job as drivers for public/ private sector companies or for Kuwaiti families.

This cross-sectional study was conducted among undergraduate students enrolled in Kuwait University for academic year 2016–17. The students who drive by themselves, of either sex, both Kuwaiti or non-Kuwaiti were eligible for inclusion in the study. In Kuwait, 17-years old or older people are eligible to seek non-professional driving license. Therefore, all the members of study population were eligible to have a driving license. This university currently is the only public-sector institution for higher education in the country. Geographically spread over six campuses, Kuwait University comprised 15 colleges, offering 76 undergraduate and 71 graduate programs with an annual enrollment of about 40,000 students of which nearly two-third are females [15].

### Data collection

**Fig. 1:**
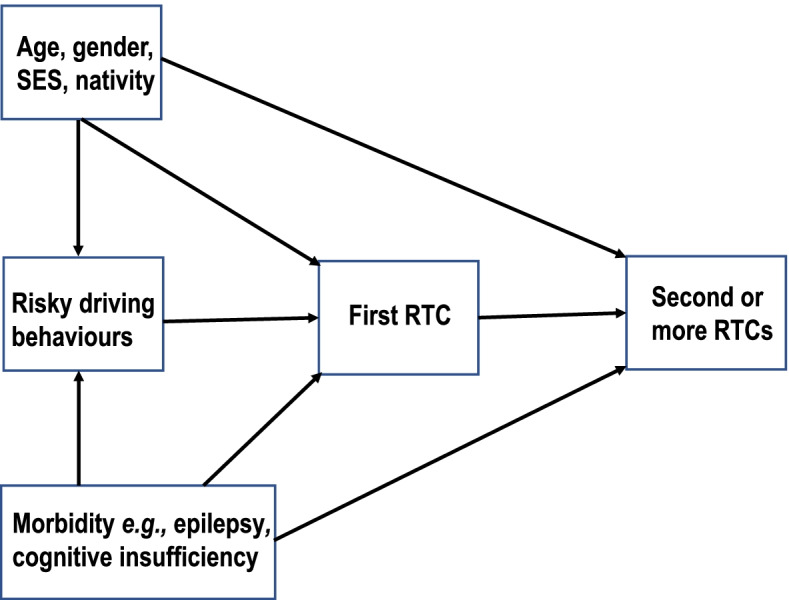
Conceptual framework of associations between sociodemographics, risk driving behaviours, prevalent morbidities and recurrent road traffic crashes among young adult drivers in Kuwait

A structured and self-administered questionnaire was developed based on literature review [16–18], and pretested before use. The questionnaire included 36 questions on potential risk factors including speeding, mobile phone use while driving, eating and or drinking etc. The questionnaire also enquired about incident road crashes during the past year and included questions regarding driving behaviors, attitudes and questions on socio-demographics. However, questions on alcohol drinking and driving were excluded because of their cultural inaptness. Outcome variable RTC was defined as an incident, involving at least one moving vehicle, that may or may not lead to injury, which occurs on a public road [19]. A conceptual framework of theoretically perceived potential associations among three sets of independent variables including demographics, risky driving behaviours, prevalent morbidities and RTCs as an ordinal outcome are given Fig. 1.

As noted earlier, the population of interest comprised a list frame of all the undergraduate students enrolled at Kuwait University for academic year 2016–17. Three interviewing teams each comprising two 5^th^ year medical students carried out the data collection. Study participants were enrolled using nonprobability convenience sampling technique both from students of science faculties (Medicine, Dentistry, Pharmacy, Allied Health, Engineering and Petroleum, Sciences, Computer Science and Engineering, Life Science, Architecture) and non-science faculties (Business Administration, Law, Literature, Education, Islamic Studies, Sharia’a, and Social sciences). The interviewers approached the students at the end of their classes or during breaks and invited them to participate in the study. Study objectives were explained to potential respondents and the consenting students were handed over the questionnaires for completion. The included participants were therefore a sample of convenience as noted earlier. The interviewers examined the filled-in questionnaires at the spot for their completeness and participants were requested to answer any questions that might have been unheeded. The power analysis and sample size computation have been reported elsewhere [4]. Briefly, *first,* to estimate 1-year RTCs prevalence in the target study population of 25 thousand of enrolled students, a sample size of 985 participants were regarded as sufficient at a 5% level of significance (α = 0.05), with 3% bound on error of estimation with assumed period prevalence (≥ 1 RTC) as 45% [18]. *Second*, to examine the potential demographics and risky behaviours associated with RTCs status, the required sample size was1100 participants (50% in each group with or without RTCs in the past one-year) under the assumptions including minimum effect size of 2 (ratio of odds), at level of significance 5% (α = 0.05), with 95% (1-ß = 0.95) study power and 10% or more prevalence of fast driving and/ or other risky driving behaviours among young drivers without RTC during the past one year [20]. Anticipated potential refusals (12%) to participate in study were are also accounted for in the final sample size estimation.

### Statistical analyses

To characterize the study sample, descriptive statistics including mean (standard deviation (SD)), or proportion (%) were computed as required. We computed one-year period prevalence (%) (95% confidence interval (CI)) of one, two, three or more RTCs during the past year. Chi-squared analysis was conducted to identify the variables significantly (*p* ≤ 0.15) related with the ordered outcome variable (*i.e.,* number of RTCs as defined above). We used the higher significance level in chi-squared analysis as it is one of the suggested approaches to relax *p*-value (*e.g.,* 0.15 or 0.2 etc.) for variable screening based on the unconditional associations of predictors with the outcome in an attempt to reducing the number of predictors for final parsimonious multivariable model selection [21, 22]. Thus, the variables that were related to the ordinal outcome in unconditional analyses were then considered for possible inclusion in multivariable analysis.

To take the advantage of the ordinality of the response variable (RTC count *i.e.,* 0,1,2, ≥ 3), an ordered logit model was considered. Ordinal regression is a more sensitive analysis than a binary logistic regression using an arbitrarily dichotomized response variable [23]. A proportional odds model—a special case of generalized ordered model that entails the parallel-lines assumption for all explanatory variables was employed. This model can be written as follow:

$$P\left({Y}_{i}>j\right)=g\left(X\beta \right)= \frac{{e}^{({\alpha }_{j}+ {X}_{i}\beta )}}{1+{e}^{\left({\alpha }_{j}+ {X}_{i}\beta \right)} }, j=1, 2, . .., J-1$$

where, *J* is the number of categories of the ordinal dependent variable. The right-hand side of model is logit defined with $P\left(Y>j\right)$ in the numerator and $P\left(Y\le j\right)$ in the denominator. A positive $\beta$ corresponds to a positive association, *i.e.,* lager values of Y are relatively more likely to occur at large X values. In this model, the regression coefficients, $\beta {\left({\beta }_{1},{\beta }_{2},\dots ,{\beta }_{p}\right)}^{^{\prime}}$ are constant across the *J* -1 cumulative logits, but not the intercepts (${\alpha }_{j})$. Thus, for each independent variable, this model presents *J* different $\alpha$ coefficients, and the same *β* coefficient for each category of ordinal response variable. This means the effect of *x* is assumed to be same for each cumulative probability; it does not depend on the cut point for forming the logit. The parameter ${e}^{\beta }$ is the multiplicative effect on the cumulative odds *i.e*. the odds that the response is greater rather than less or equal to *j* (for any fixed *j*) is multiplied by ${e}^{\beta }$ for every unit change in x [24, 25]. Furthermore, it allows for the appraisal of the homogeneity of the odds ratios over various levels of response variable [26].

First, for each of the independent variables identified as significantly related to the ordered outcome in chi-squared analysis, simple proportional odds model was fitted to quantify its unadjusted strength of association with RTC (ordered response variable as defined earlier). Subsequently, a full model including all main effects was specified and evaluated. A backward stepwise modeling procedure was adapted for final model selection. Log-likelihood ratio test and Akaike information criterion (AIC) statistics were used to compare the nested and competing models. Additionally, as noted earlier, all the predictors considered in proportional odds model essentially need to satisfy the proportional odds (PO) assumption *i.e.,* each predictor has the same effects across the categories of the ordinal outcome variable, which can be judged whether logit regression coefficients for each predictor are the same across the ordinal categories of the outcome. To test whether PO assumption is met, Brant test was used to assess the PO assumption for each predictor univariably and with the global Brant test for all predictors together in the overall model [27]. All the analysis were carried out with Stata 16.1/ SE (Stata Corp LLC).

## Results

The study participants were selected from 15 faculties/ colleges of Kuwait University. A total of 1500 students were approached and invited to participate in the study. Of the invitees, 1465 consented and completed the questionnaire with an overall response rate of 97.6% (1465/1500). Among the non-respondents (*n* = 35) cited reasons for their non-participation were ‘very busy’ [24] or ‘not interested’ [11]. The majority (24.0%) came from the Faculty of Medicine. Of the participants, majority (67.1%) were Kuwaiti nationals, females (56.4%) with an age range of 17–38 years (Table 1).

**Table 1:** Socio-demographic characteristics of the university students enrolled in a study of one-year period prevalence of road traffic crashes in Kuwait, December 2016 (*N* = 1465)

*Demographic characteristics*	Total study sample (*n* = 1465)^a^	%
Age (years), n (%)%		
17–20	537	37.7
21–25	804	56.4
≥ 26	85	6.0
Missing, n	39	2.7
Age (years), Mean (SD); Range (min – max)	21.5 (3.3) (17–38)	
Gender, n (%)		
Male	417	28.5
Female	1046	71.5
Missing, n	2	0.1
Nationality		
Kuwaiti	980	67.1
Non-Kuwaiti	480	32.9
Missing, n	5	0.3
Governorate of residence		
Capital	348	23.8
Hawalli	389	26.6
Farwaniya	204	13.9
Mubarak Al-Kabeer	171	11.7
Ahmadi	162	11.1
Jahra	189	12.9
Missing, n	2	0.1
Faculty/ College of studies^b^		
Science faculties	857	59.2
Non-Science faculties	596	40.8
Missing, n	3	0.2

^a^Category totals may not add up to total because of missing values

^b^Science Faculties (Medicine, Dentistry, Pharmacy, Allied Health, Engineering and Petroleum, Sciences, Computer Science and Engineering, Life Science, Architecture); Non-Science Faculties (Business and Administration, Law, Literature, Education, Islamic Studies and Sharia’a, Social sciences

In this study population, one-year period prevalence (%) of having one, two, three or more RTCs was respectively 23.1 (95% CI: 21.2, 25.6), 10.9 (95% CI: 9.4,12.6) and 4.6 (95% CI: 3.6, 5.9) (Table 2). Of the participants 171 (11.7%) reported to have suffered either minor (142, 9.7%) or severe injury that required ambulance (29, 2.0%) during RTC. Body sites affected during the RTC included head and neck (48, 3.3%), back (29, 2.0%), pelvis (6, 0.4%) and limbs (arms and legs) (88, 6.0%).

**Table 2:** Chi-squared analysis of the relationship between demographic characteristics and prevalence of road traffic crashes (RTC) among university students in Kuwait, December 2016 (*N* = 1465)

Characteristic^d^	Distribution of number of RTCs^b^				*P*-value
	None *n* = 895 (61.1%)	One *n* = 342 (23.3%)	Two *n* = 160 (10.9%)	Three or more *n* = 68 (4.6%)	
Age (completed years)					0.413^a^
17–20	339 (63.8)	107 (20.2)	60 (11.3)	25 (4.7)	
21–25	470 (58.8)	200 (25.0)	87 (10.9)	43 (5.4)	
26 and more	52 (61.9)	21 (25.0)	9 (10.7)	2 (2.4)	
Gender					0.516
Male	242 (58.6)	105 (25.4)	43 (10.4)	23 (5.6)	
Female	640 (61.7)	232 (22.3)	117 (11.3)	49 (4.7)	
Nationality					
Kuwaiti	592 (60.9)	225 (23.1)	112 (11.5)	44 (4.5)	0.524
Non-Kuwaiti	289 (60.7)	113 (23.7)	46 (9.7)	28 (5.9)	
Governorate					0.127
Capital	202 (58.1)	90 (25.9)	41 (11.8)	15 (4.3)	
Hawali	248 (63.9)	94 (24.2)	36 (9.3)	10 (2.6)	
Farwaniya	119 (59.2)	44 (21.9)	21 (10.5)	17^b^.5)	
Mubarak Al-Kabeer	98 (58.0)	43 (25.5)	20 (11.8)	8 (4.7)	
Ahmadi	97 (61.0)	28 (17.6)	21 (13.2)	13 (8.2)	
Jahra	119 (63.7)	38 (20.3)	21 (11.2)	9 (4.8)	
Faculty/ Colleges of studies^c^					0.981
Science faculties	517 (60.3)	202 (23.6)	95 (11.1)	43 (5.0)	
Non-Science faculties	366 (61.4)	136 (22.8)	65 (10.9)	29 (4.9)	

^**a**^Fisher Freeman-Halton exact test

^b^Category totals may not add up to total because of missing values

^c^Science Faculties (Medicine, Dentistry, Pharmacy, Allied Health, Engineering and Petroleum, Sciences, Computer Science and Engineering, Life Science, Architecture); Non-Science Faculties (Business and Administration, Law, Literature, Education, Islamic Studies and Sharia’a, Social sciences

^d^Data on missing information on the variables have been provided on previous Table 1

Chi-squared analysis of multiway contingency table revealed that barring governorate of residence, none of the other demographics including age, gender, nationality, and college of enrollment was statistically significantly (*p* > 0.15) associated with the ordinal RTCs variable (Table 2). However, the driving behaviors, that were significantly (*p* ≤ 0.15) associated with the ordinal RTC status as a dependent variable included habitually speeding over limit, using a hand-held mobile phone while driving, wearing a seatbelt, eating/drinking while driving, smoking while driving, habitually running through a red light, driving on emergency lanes, number of speeding tickets during past one year, number of parking tickets during past one year, and frequency of parking in a no parking zone. However, age (years) at first started driving and average number of hours of daily sleep were not significantly (*p* > 0.15) related with the ordinal outcome variable. Furthermore, of the comorbid conditions considered including vision and/or hearing problems, asthma, migraine, or any other self-rated medical condition, epilepsy was the only morbid condition that was significantly (*p* = 0.015) associated with the ordinal RTC status on chi-squared analysis (Table 3).

**Table 3:** Chi-squared analysis of the relationship between drivers’ behaviors during driving and one-year period prevalence of road traffic crashes among university students in Kuwait, December 2016 (*N* = 1465)

Driver’s behavior’s during driving^b^	Distribution of number of road traffic crashes^a^				*P*-value
	None (*n* = 899, 61.4%)	One (*n* = 338, 23.1%	Twice (*n* = 160; 10.9%)	Thrice or more (*n* = 68; 4.6%)	
	n (%)^a^	n (%)^a^	n (%)^a^	n (%)^a^	
Age (years) at first started driving (< 18/ ≥ 18)					0.219
≥ 18	707 (60.8)	276 (23.7)	129 (11.1)	51 (4.4)	
< 18	175 (60.6)	62 (21.5)	31 (10.7)	21 (7.3)	
Ever speed over limit for the road (y/n)					< 0.001
Yes	373 (54.9)	161 (23.7)	100 (14.7)	46 (6.8)	
No	506 (65.9)	176 (22.9)	60 (7.8)	26 (3.4)	
Use phone when driving (y/n)					0.005
Yes	461 (56.8)	200 (24.7)	103 (12.7)	47 (5.8)	
No	417 (65.5)	138 (21.7)	57 (9.0)	25 (3.9)	
Regularly wear seatbelt while driving (y/n)					< 0.001
Yes	606 (64.3)	200 (21.2)	103 (10.9)	34 (3.6)	
No	275 (54.2)	138 (27.2)	57 (11.2)	37 (7.3)	
Eat/drink while driving (y/n)					0.003
Yes	519 (57.4)	221 (24.4)	116 (12.8)	49 (5.4)	
No	362 (66.4)	116 (21.3)	44 (8.1)	23 (4.2)	
Smoking while driving (y/n)					0.052
Yes	80 (51.0)	47 (29.9)	19 (12.1)	11 (7.0)	
No	800 (61.9)	291 (22.5)	141 (10.9)	61 (4.7)	
Habitually crossed a red light (y/n)					< 0.001
Yes	273 (51.2)	139 (26.1)	77 (14.5)	44 (8.3)	
No	605 (66.1)	199 (21.8)	83 (9.1)	28 (3.1)	
Drive on emergency lane (y/n)					0.005
Yes	304 (56.3)	135 (25.0)	62 (11.5)	39 (7.2)	
No	576 (63.3)	203 (22.3)	98 (10.8)	33 (3.6)	
Speeding tickets during the past 1-year					< 0.001
None	422 (65.0)	151 (23.3)	55 (8.5)	21 (3.2)	
One	210 (57.9)	85 (23.4)	41 (11.3)	27 (7.4)	
Two	183 (62.2)	68 (23.1)	31 (10.5)	12 (4.1)	
Three or more	65 (45.5)	34 (23.8)	33 (23.1)	11 (7.7)	
Parking tickets in the past 1-year					< 0.001**
None	441 (84.6)	54 (10.4)	20 (3.8)	6 (1.2)	
One	130 (30.4)	179 (41.9)	80 (18.7)	38 (8.9)	
Two	77 (65.8)	67 (33.0)	43 (21.2)	16 (7.9)	
Three or more	109 (42.6)	92 (35.9)	37 (14.5)	18 (7.0)	
Parked in the no parking zone					0.006**
None	174 (68.5)	54 (21.3)	20 (7.9)	6 (2.4)	
1–5	474 (61.5)	179 (23.2)	80 (10.4)	38 (4.9)	
6–10	166 (56.8)	67 (23.0)	43 (14.7)	16 (5.5)	
More than 10	65 (49.2)	38 (28.8)	17 (12.9)	12 (9.1)	
Average hours of sleep every night					0.582**
5 or less	176 (62.2)	54 (19.1)	35 (12.4)	18 (6.3)	
6–8	533 (60.5)	209 (23.7)	97 (11.0)	42 (4.8)	
8–10	136 (61.0)	56 (25.1)	23 (10.3)	8 (3.6)	
More than 10	37 (56.9)	18 (27.7)	5 (7.7)	5 (7.7)	
Being a patient with epilepsy					
No	723 (62.1)	267 (22.9)	127 (10.9)	48 (4.1)	0.015***
Yes	3 (23.1)	5 (38.5)	2 (15.4)	3 (23.1)	

^**^ *p*-value for with Chi-squared statistic for trend

^***^ Goodman–Kruskal gamma test of independence

^a^Category totals may not add to total due to missing values

^b^The missingness for the variables list this table ranges from 0.0% to 0.8% except epilepsy which was 19.6% most like answer would have been no. (age (years) first started driving = 7 (0.5%); Ever speed over limit for the road = (0.8%); use phone while driving = 12 (0.8%); Regularly wear seatbelt while driving = 10 (0.7%); Ear/ drink while driving = 10 (0.7%); Smoking while driving = 10 (0.7%); Habitually crossed red light = 1 (0.8%); Drive in emergency line = 10 (0.7%); Number of speeding tickets during the past 1 year 11 (0.8%); Number of parking tickets during the past one year = 0 (0.0%; Number of times parked in no parking zone = 11 (0.8%); Hours of sleep every night = 8 (0.5%); suffer from epilepsy = 287 (19.6%), most likely to ‘no’ answer

The results of the multivariable proportional odds model are in Table 4. The Brant test statistic was insignificant (*p* > 0.150) for each of the predictors individually as well as in global Brant test (*p* = 0.189) for the variables in the model indicating that proportional odds assumption was satisfied by each predictor individually as well as all the predictors together in the final model. The log likelihood ratio chi-squared test statistic (LR ${\upchi }_{df=9}^{2}$= 80.89) was significant (*p* < 0.001), which indicated that the full model with five predictors provided a better fit than the null model in predicting the ordinal response variable. Final multivariable proportional odds model revealed that the participants tended to have a number of RTCs above as opposed to equal or below their current RTC count status, if they habitually violated the speed limit (adjusted proportional OR (pOR_adjusted_) = 1.40; 95%CI: 1.13,1.75; *p* = 0.002), habitually ran through the red light (pOR_adjusted_ = 1.64; 95%CI: 1.30, 2.06; *p* < 0.001), had speeding ticket three or more times during the past year (pOR_adjusted_ = 1.63; 95%CI: 1.12, 2.38; *p* = 0.011) or habitually parked in no-parking zone more than ten times during the past year (pOR_adjusted_ = 1.64; 95%CI: 1.06, 2.54; *p* = 0.027). Similarly, being a patient with epilepsy made it more likely for the participant to encounter higher number of RTCs than their current or less RCTs count (pOR_adjusted_ = 4.37; 95%CI: 1.63,11.70; *p* = 0.003).

**Table 4:** Multivariable proportional odds model of high-risk drivers’ attributes associated with road traffic crashes in Kuwait (N = 1447)

Characteristics	Crude pOR (95% CI)	% (n / total)	Adjusted pOR (95% CI)	*P*—value
Habitually violated of speed limit (yes/no)	1.73 (1.41 – 2.13)	46.9 (681/1453)	1.40 (1.13 –1.75)	0.002
Habitually crossed red light (yes/ no)	2.00 (1.62 – 2.47)	36.8 (534/453)	1.64 (1.30 – 2.06)	< 0.001
Speeding ticket (vs. none)				
Once	1.48 (1.15 – 1.91)	25.0 (364/454)	1.10 (0.83 – 1.45)	0.523
Twice	1.16 (0.88 – 1.53)	20.3 (295/1454)	1.01 (0.75 – 1.35)	0.958
Thrice or more	2.56 (1.81 – 3.62)	9.8 (43/454)	1.63 (1.12 – 2.38)	0.011
Parked in no parking zone (vs. none)				
1–5	1.40 (1.04 –1.88)	53.2 (774/1454)	1.30 (0.96 –1.76)	0.095
6–10	1.79 (1.27 – 2.52)	20.2 (293/454)	1.45 (1.00 – 2.09)	0.049
More than 10	2.36 (1.56 – 3.57)	9.1 (132/1454)	1.64 (1.06 – 2.54)	0.027
Being epilepsy patient (yes/no)	4.60 (1.72 – 12.34)	1.1 (13/1178)	4.37 (1.63 – 11.70)	0.003

*pOR* Proportional odds ratio, *CI* Confidence interval

## Discussion

Reportedly, young drivers have a propensity for recurrent RTCs within a year [11]. The identification of attributes of such young drivers with inclination to repeated RTCs is a prerequisite for the formulation of a focused educational program. Therefore, in this analysis, the number of RTCs was considered as an ordinal outcome with ordered categories (*i.e.,* 0,1,2, ≥ 3 RTCs) rather than a binary dependent variable for the use of conventional binary logistic regression. Additionally, when the prediction of an ordinal outcome with respect to several independent variables is of interest, proportional odds or partial proportional odds modeling is a preferred analytic technique. The choice of either of these two models is contingent on whether or not the putative predictors of an ordinal outcome meet the assumption of proportionality of the odds [28]. Moreover, it has been shown that collapsing the categories of an ordinal dependent variable to reduce it to a binary variable for the application of binary a logistic regression model leads to the loss of efficiency [13].

This study assessed 1-year period prevalence of one, two or three or more RTCs and examined the risk behaviours associated with this ordinal outcome (*i.e.,* none, one, two or three or more RTCs) among young adult drivers in Kuwait. In the study sample, 1-year period prevalence (%) of having one, two, three or more RTCs respectively was 23.3, 10.9 and 4.6. Thus,15.5% of young adult drivers had repeated RTCs during the past one year. In Louisiana, USA, 34% of RTCs were repeatedly committed by the at-fault crash-prone drivers who represented only 5% of the total licensed drivers [29]. This difference between two studies on the proportions (15.5% vs. 34.0%) of crash-prone drivers having more than one crashes seem to be due to periods (1 vs. 2 years) covered. In Egypt, 25% of the medical students had multiple RTCs [30], a proportion somewhat higher than 15.5% recorded in this study. RTC-prone drivers get into recurring crashes that are caused by human error and/ or driving behaviours rather than just by coincidence [31]. The high magnitude of prevalence estimates for repeated RTCs signifies the gravity of the problem in this young driving population.

### Risk behaviors associated ordinal outcome (i.e., one, two or three and more RTCs)

A tendency of violating the speed limit was significantly associated with increased odds of being in a higher category of RTC count than being in their current or lower RTCs category in this study. In Qatar, 36.9% of the drivers who routinely exceeded speed limits have had multiple RTCs within a year [32]. Another study showed that the individuals with crash history were at higher risk of having subsequent crash, who routinely violated the speed limit [33]. Therefore, enforcement of traffic laws that discourage the speeding are likely to reduce the RTCs risk and potential injuries and/ or deaths in this and other similar settings.

In this study, respondents who have had three or more speeding tickets during the past year had increased likelihood to be in a higher RTCs count category than being in their current or lower RTCs’ count status. An ecological study in Arabian GCC countries demonstrated that increasing number of citations on excessive speed was significantly associated with future RTCs episodes [34]. Another Middle-Eastern study reported that the probability of involvement in subsequent RTC was more than eleven times higher for drivers with six traffic tickets per year compared to those with none or one ticket per year [35]. It has been shown that the citations on speeding among repeat traffic laws offenders have limited deterrence effect in the context of traffic laws violation, thus such offenders continue to be at higher risk of future RTCs [36]. Identification and targeted educational program of such traffic laws offenders is warranted, which if implemented is likely to pay dividends in terms of RTCs reduction and associated losses.

Respondents with history of red traffic light violation tended to be in higher RTCs count category than being in their current or below RTCs count in this study. Failing to stop at a red light has been identified a specific traffic law offence that was associated with repeated and severe crashes [37]. In Egypt, 25% of medical students who used to frequently disregard traffic lights and with a history of previous crash had subsequent RTC [30]. Running through the red light is a serious traffic offence that entailed explicit education and subsequent imposition of heavy penalty on violators of this law.

In this study, we found that the respondents who repeatedly ignored the no parking zone signs while parking their vehicles during the past year were significantly more likely to have higher RTCs count than being in their current or low RTCs frequency. A recent meta-analysis revealed that the individuals who habitually and frequently commit non-moving traffic offences were more likely to have repeated RTCs [38]. Such young drivers in this and other similar settings in the region need to be educated to strictly adhere to traffic signs. Therefore a focused educational program may help in behaviour modification concerning overall traffic laws.

We found that respondents with history of epilepsy diagnosis were significantly more likely to be in a higher RTCs count category than being in current or lower RTC count group. To the best of our knowledge, there are no published data that specifically examined the association between being a patient with epilepsy and recurrent RTCs risk among young drivers. However, a study in India showed that the characteristics associated with multiple crashes in RTCs-prone drivers included medical conditions [39]. Drivers with intermittent epileptic seizures are at high risk of RTCs due to the nature of the disease, which involves involuntary movements, impaired consciousness caused by epileptic seizures and side effects of antiepileptic drugs [40]. Issuing of driving license to individuals with epilepsy should be contingent on neurologists’ certification of their ability to drive safely and seizure-free for a specific period of time and/or periodic submission of medical reports as practiced in many developed countries [41–44]. The enactment of similar laws in this and other comparable settings in the region may enhance the road safety and minimize the frequency of recurrent RTCs.

### Strengths and limitations

The key strength of this study is the application of the multivariable proportional odd model in RTCs epidemiology, which may motivate the investigators in medicine and other allied fields to employ this or related models developed for ordinal outcomes. Following limitations of this study need to be considered in the interpretation of the results; First, we could not enroll students from the private-sector universities in Kuwait because of semester break. Therefore, we had to limit our data collection from the students enrolled in Kuwait University—the only public-sector university. Nevertheless, we believe that the students enrolled in private universities in Kuwait would be similar in driving behaviours and practices as our participants with nearly uniform demographic characteristics such as age, nationality etc. However, future studies on this topic may consider expanding the scope of their sampling across the whole spectrum of institutions; Second, recall bias might have crept in the collected data because of self-reporting inaccuracies, which could have led to an under-estimation of one-year period prevalence of RTCs count and/or effect size for risk behaviors in relation to RTCs in the multivariable proportional odds model. However, we presume that this bias should be minimal because the reporting period was only one-year which might have not considerably hampered the recall; Third, the participants completed the questionnaire, which were checked by the interviewers in front of the respondents. The likelihood of social desirability bias in responses would have been a possibility. Because data collection team checked the returned questionnaires for their completion at the spot. Therefore, this potential social desirability could have resulted in somewhat underestimation of RTC counts’ period prevalence and/ or effect size of evaluated risk behaviours [45]. Nonetheless, we believed that the extent of such a bias would be minimal because the interviewers were respondents’ peers who themselves were likely to engage in similar behaviours and Final, the study participants were selected as a sample of convenience, therefore care needs to be exercised in generalizing the results beyond the study sample. However, though the study sample was statistically non-representative of all the young adults of this age bracket in Kuwait but was typical of this reference population. Therefore, we feel somewhat contented regarding the external validity of the results of this study.

## Conclusions

This study revealed that during the past one-year, ensuing the first RTC incident,15.5% of the young adult drivers tended to encounter second, or third or more RTCs during the same year. Young adult drivers who habitually violated speed limit, had received multiple citations on speeding, routinely ran through the red light, frequently parked in no-parking zone and/ or being a patient with epilepsy have had the propensity for multiple RTCs during the past one year. Education of young adult drivers with focus on identified risky driving behaviours, and subsequently imposing heavy penalties on traffic laws offenders including suspension of driving license for a stipulated period may help modification of risky driving behaviours and curtailment of recurring RTCs in this and other similar settings. Furthermore, the validity period of driving license for patients with epilepsy should be constrained with the physician certification of their ability to drive safely and seizure free along with the submission of periodic medical reports as is being practiced elsewhere [46, 47]. If effected, future studies may look at the impact of proposed interventions.

## Data Availability

The dataset supporting the conclusions of this article can be made available on reasonable request after the approval by the authors.

## Abbreviations

RTCs: Road traffic crashes
CI: Confidence interval;
pOR_adjusted_: Adjusted proportional odds ratio
GDP: Gross Domestic Product
WHO: World Health Organization
SD: Standard deviation
AIC: Akaike information criterion
LR ${\upchi }_{df}^{2}$: Log likelihood ratio chi-squared test statistic
USA: United States of America
GCC: Gulf Cooperation Council
